# The concept that focuses on oral motor and feeding function in cancer patients with muscle wasting: Skeletal muscle mass is associated with severe dysphagia in cancer patients

**DOI:** 10.1002/jcsm.12119

**Published:** 2016-05-02

**Authors:** Masakazu Saitoh, Junichi Ishida, Masaaki Konishi, Jochen Springer

**Affiliations:** ^1^ Innovative Clinical Trials, Department of Cardiology and pneumologyUniversity Medical Center GöttingenGöttingenGermany

I read with great interest the recent article reporting the relation between skeletal muscle mass and dysphagia in cancer patients by Hidetaka Wakabayashi et al.^1^ In criteria for the diagnosis of sarcopenia, loss of muscle mass, reduced handgrip strength, or gait speed is assessed as representative value of impaired muscle strength or physical performance.^2^, ^3^ Assessment of oral motor and feeding function is not common in diagnostic testing for sarcopenia or cachexia; however; loss of muscle mass is supposed to be spread throughout the entire body.

Insufficient dietary intake and decreased appetite are major factors for loss of muscle mass.^4^ Therefore, increasing dietary intake and appetite could be one of the treatments for improvement of muscle wasting. However, some complications of cancer or chemotherapy often interfere with appetite, oral motor and feeding function in cancer patients.^5^, ^6^ Loss of muscle mass and impaired physical performance, and activity of daily living (ADL) induced by cancer and chemotherapy cannot be fully reversed by conventional nutritional support alone.^7^, ^8^ These clinical trials support the hypothesis that nutritional support would not be enough for strategy in advanced cancer patients with of oral motor and feeding dysfunction. In order to make a better understanding the strategy of cancer patients with sarcopenia or cachexia, evaluation of the oral motor and feeding function is no less important than assessment of dietary habit or nutritional status according to biochemical, anthropometric measures, and nutritional screening tool. It remains unclear whether hybrid strategy combining both nutritional support and exercise or rehabilitation affected on the loss of muscle mass, impaired ADL, and quality of life in cancer patients with dysphagia accompanied by sarcopenia or cachexia.^4^, ^9^ Further advances are required.

## Acknowledgements

We acknowledge support by the German Research Foundation and the Open Access Publication Funds of the Göttingen University. The authors certify that they comply with the ethical guidelines for publishing in the Journal of Cachexia, Sarcopenia and Muscle: update 2015.^10^

## Conflicts of interest

M. Saitoh, J. Ishida, M. Konishi, and J. Springer declare that they have no conflict of interest.

## References

[1] Wakabayashi H , Matsushima M , Uwano R , Watanabe N , Oritsu H , Shimizu Y . Skeletal muscle mass is associated with severe dysphagia in cancer patients. J Cachexia Sarcopenia Muscle 2015;6:351–7. 26673551 10.1002/jcsm.12052 PMC4670744

[2] Cruz‐Jentoft AJ , Baeyens JP , Bauer JM , Boirie Y , Cederholm T , Landi F , Martin FC , Michel JP , Rolland Y , Schneider SM , Topinková E , Vandewoude M , Zamboni M . European Working Group on Sarcopenia in Older People. Sarcopenia: European consensus on definition and diagnosis: Report of the European Working Group on Sarcopenia in Older People. Age Ageing 2010;39:412–23. 20392703 10.1093/ageing/afq034 PMC2886201

[3] Morley JE , Cao L . Rapid screening for sarcopenia. J Cachexia Sarcopenia Muscle 2015;6:312–4. 26676168 10.1002/jcsm.12079 PMC4670738

[4] Anker SD , Morley JE . Cachexia: a nutritional syndrome? J Cachexia Sarcopenia Muscle 2015;6:269–71. 26675043 10.1002/jcsm.12088 PMC4670732

[5] Dysphagia Section, Oral Care Study Group, Multinational Association of Supportive Care in Cancer (MASCC)/International Society of Oral Oncology (ISOO) , Raber‐Durlacher JE , Brennan MT , Verdonck‐de Leeuw IM , Gibson RJ , Eilers JG , Waltimo T , Bots CP , Michelet M , Sollecito TP , Rouleau TS , Sewnaik A , Bensadoun RJ , Fliedner MC , Silverman S Jr , Spijkervet FK . Swallowing dysfunction in cancer patients. Support Care Cancer 2012;20:433–443. 22205548 10.1007/s00520-011-1342-2 PMC3271214

[6] Vaughan VC , Martin P , Lewandowski PA . Cancer cachexia: impact, mechanisms and emerging treatments. J Cachexia Sarcopenia Muscle 2013;4:95–109. 23097000 10.1007/s13539-012-0087-1 PMC3684701

[7] Aapro M , Arends J , Bozzetti F , Fearon K , Grunberg SM , Herrstedt J , Hopkinson J , Jacquelin‐Ravel N , Jatoi A , Kaasa S , Strasser F ; ESMO (European School of Medical Oncology). Early recognition of malnutrition and cachexia in the cancer patient: a position paper of a European School of Oncology Task Force Ann Oncol 2014; 25: 1492‐9. 24569913 10.1093/annonc/mdu085

[8] Omlin A , Blum D , Wierecky J , Haile SR , Ottery FD , Strasser F . Nutrition impact symptoms in advanced cancer patients: frequency and specific interventions, a case‐control study. J Cachexia Sarcopenia Muscle 2013;4:55–61. 23307589 10.1007/s13539-012-0099-x PMC3581613

[9] Bowen TS , Schuler G , Adams V . Skeletal muscle wasting in cachexia and sarcopenia: molecular pathophysiology and impact of exercise training. J Cachexia Sarcopenia Muscle 2015;6:197–207. 26401465 10.1002/jcsm.12043 PMC4575550

[10] von Haehling S , Morley JE , Coats AJS Anker SD . Ethical guidelines for publishing in the Journal of Cachexia, Sarcopenia and Muscle: update 2015. J Cachexia Sarcopenia Muscle 2015;6:315–316. 26672494 10.1002/jcsm.12089 PMC4670739

